# Prevalence of *Toxoplasma gondii* Antibodies and Risk Factors in Two Sympatric Invasive Carnivores (*Procyon lotor* and *Nyctereutes procyonoides*) from Zgorzelec County, Poland

**DOI:** 10.3390/pathogens13030210

**Published:** 2024-02-28

**Authors:** Natalia Osten-Sacken, Jutta Pikalo, Peter Steinbach, Mike Heddergott

**Affiliations:** 1 Centre for Veterinary Sciences, Nicolaus Copernicus University, 87-100 Toruń, Poland; 2 Institute of Parasitology, University of Veterinary Medicine Vienna, Veterinärplatz 1, 1210 Vienna, Austria; jutta.pikalo@vetmeduni.ac.at; 3 Department of Zoology, Musée National d’Histoire Naturelle, 2160 Luxembourg, Luxembourg; psteinbach@web.de (P.S.); mike-heddergott@web.de (M.H.); 4 Faculty of Chemistry, Georg-August University of Göttingen, 37073 Göttingen, Germany

**Keywords:** *Toxoplasma gondii*, modified agglutination test (MAT), raccoon, *Procyon lotor*, raccoon dog, *Nyctereutes procyonoides*, invasive species, wildlife, serological detection, zoonotic

## Abstract

The intracellular protozoan *Toxoplasma gondii* is distributed worldwide and infects many species of warm-blooded animals. Most mammals, including humans, can serve as intermediate hosts. This pathogen, with its zoonotic potential, causes toxoplasmosis, a condition that can range from subclinical to fatal in humans. It is therefore important to assess the occurrence of the pathogen, even if only indirectly through the detection of antibodies. Epidemiological data on the seroprevalence in wild animals, including invasive species, are rare in Poland. Therefore, we tested 197 wild raccoons (*Procyon lotor*) and 89 raccoon dogs (*Nyctereutes procyonoides*) from Zgorzelec County, southwestern Poland, for the presence of antibodies. Samples were collected between January 2019 and December 2020 and analysed using a commercial indirect modified agglutination test (MAT, cut-off 1:25). The statistical analysis revealed significant differences in seroprevalence between the two predatory species. Of the 197 surveyed raccoons, 96 (48.73%; 95% confidence interval (CI): 41.73–55.73%) tested positive, while 25 of the 89 raccoon dogs (28.09%; 95% CI: 18.70–37.48%) were positive. Regarding risk factors, body weight and sex influenced the presence of *T. gondii* antibodies in both the species, with a higher likelihood of seropositivity among heavier animals and females, respectively. For raccoon dogs, juveniles were more likely to be seropositive than adults at a given weight. Our results suggest that *T. gondii* infection is widespread in the regional raccoon and raccoon dog populations, indicating a high level of parasite circulation in the environment.

## 1. Introduction

*Toxoplasma gondii* is a zoonotic, globally distributed, obligatory intracellular protozoan. It infects a variety of warm-blooded vertebrates, including humans. It has been estimated that one-third of the world’s population is infected with this parasite [1]. *T. gondii* has an indirect life cycle in which domestic and wild cats are the final hosts and the oocysts are excreted into the environment via feces. In addition to vertical transmission (i.e., from mothers to their offspring), infection occurs mainly via the faecal–oral route. The infection is caused by the intake of water or food contaminated with sporulated *T. gondii* oocysts or by the consumption of tissues from animals infected with encapsulated tissue cysts (bradyzoites) [1].

The raccoon (*Procyon lotor*), a North and Central American mesocarnivore of the Procionidae family, has been introduced for fur breeding and hunting and as a pet in several European and Asian countries over the last century [2,3,4]. The raccoon is particularly widespread in Germany, expanding into neighbouring countries as its geographical range has increased in recent decades [5]. Genetic studies have shown that German raccoons originated from various distinct founding events [6,7]. The first records of raccoons in Poland date from the middle of the 20th century, while established populations were not recorded until the 1990s. Raccoons from two genetic clusters located in eastern Germany have been spreading to adjacent areas in Poland [8] and the number of raccoons in Poland has increased in recent decades [9].

The raccoon dog (*Nyctereutes procyonoides*) is a member of the Canidae family whose original area of distribution in East Asia includes eastern Siberia, China, the Korean peninsula and Japan [10]. During the 1930s and 1950s, some 10,000 animals were translocated from the eastern to the western part of the former Soviet Union. Initially, this was for fur farming purposes, later for deliberate releases [11]. Further populations were established in the Ukraine and Belarus with animals stemming from these first introductions [12]. After these successful introductions, the raccoon dog rapidly extended its range [13]. Currently, the species is widespread in Eastern and Northern Europe and is spreading further into Central Europe [11,14]. In Poland, the raccoon dog is the most widespread invasive carnivore species. It was first recorded in the first half of the 1950s in eastern Poland. The species subsequently spread westwards and, with the exception of a few mountainous areas, colonized the entire country by the end of the 1970s [9].

Because of their omnivorous lifestyles, raccoons and raccoon dogs can act as hosts for numerous zoonotic pathogens [7,15,16,17,18,19,20,21,22,23,24,25,26,27]. In general, there are only a few studies on *T. gondii* infection in raccoons and raccoon dogs in Poland [28,29,30,31]. Here, we report the seroprevalence of *T. gondii* observed in raccoons and raccoon dogs from Zgorzelec County in southwestern Poland and identify factors that determine infection prevalence. We hope that our findings will help to understand the extent of environmental contamination and spread of *T. gondii* in the ecosystem of this region.

## 2. Materials and Methods

### 2.1. Ethical Statement

The raccoon and raccoon dog are listed as invasive species in Poland and are not protected by law. Licensed hunters can harvest both the species outside the closed season and without a special permit. All animals were legally shot and made available to the authors. No animals were killed for the purpose of providing samples for this study.

### 2.2. Sample Collection

**Figure 1:**
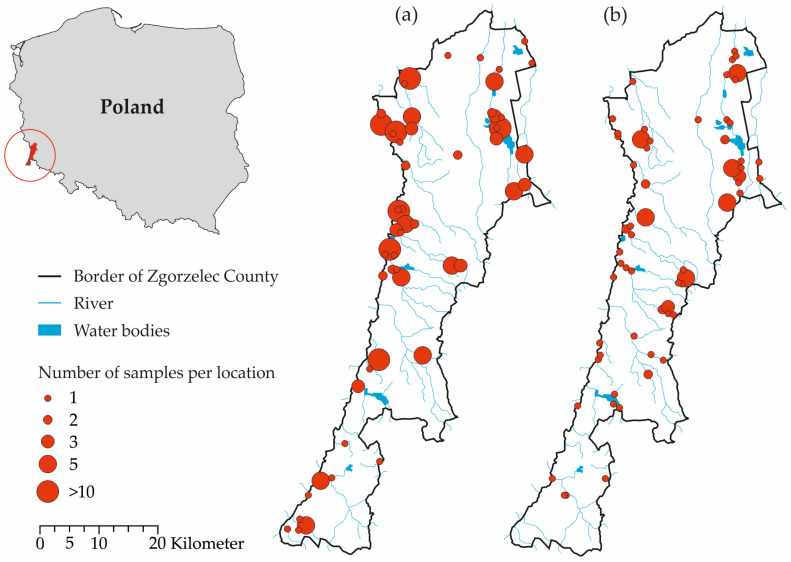
Geographical origin of the samples included in the study from Zgorzelec County in southwestern Poland. (**a**) Raccoon (*Procyon lotor*). (**b**) Raccoon dog (*Nyctereutes procyonoides*). The size of the circle indicates the number of samples analysed per location.

Between January 2019 and October 2020, legally shot or road-killed raccoons (*n* = 197) and raccoon dogs (*n* = 89) were collected in Zgorzelec County and the geographical origin of each animal was recorded (Figure 1). Zgorzelec County (50°51′–51°27′ N and 14°59′–15°11′ E) has an area of 838 km^2^ and is located in the province of Lower Silesia in southwestern Poland, bordering both Germany and the Czech Republic (Figure 1). Seventy of the raccoons examined here were included in an earlier study on infection with *Pearsonema* spp. [32].

During dissection, blood samples were taken from the heart or chest cavity and then centrifuged at 1000× *g* for 15 min using an EBM 200 tabletop centrifuge (Hettich GmbH & Co. KG, Tuttlingen, Germany). The obtained serum was stored at −20 °C until further analysis [33]. Individuals were separated into two age classes based on the incremental growth lines in the cementum of a mandibular canine: individuals without growth lines were categorised as juveniles and individuals with one or more growth lines as adults [34]. We recorded the sex and weight of each animal. The raccoons consisted of 100 males and 97 females as well as 116 adults and 81 juveniles. The raccoon dogs comprised 49 males and 40 females, as well as 39 adults and 50 juveniles.

### 2.3. Detection of T. gondii Antibodies

Sera were tested for antibodies against *T. gondii* with the indirect modified agglutination test (MAT) using a commercial kit (Toxo-Screen DA^®^, bioMérieux, Lyon, France) including negative and positive control samples. We tested with a dilution of 1:25, 1:50, 1:100 and 1:500 for the determination of *T. gondii* antibodies. A cut-off titre of 1:25 was chosen to maximise the sensitivity and specificity of the test [35]. Sera with a titre of 1:25 or higher were considered positive and those with a questionable result were retested [33,36].

### 2.4. Statistical Analysis

We calculated the 95% confidence interval (CI) for the prevalence estimates using the “Wilson” score interval method [37]. We used a *χ*^2^-test to test for differences in prevalence between both the species. We fitted logistic regressions with linear mixed-effects models (GLMMs) in the glmmTMB package [38] in order to test for an effect of weight, sex, age and collection year on the presence *T. gondii* antibodies in each species. We estimated the variation inflation factors (VIFs) with the full GLMMs without interactions to test for multicollinearity. We deemed that the independent variables exhibited no significant correlation when VIF values were <5 [39]. We only considered two-way interactions to avoid convergence and fitting issues and to facilitate interpretation. We used the dredge() function in the MuMInv.1.46.0 R package [40] to estimate Akaike information criterion (AICc) values for all potential models and selected those models whose AICc values were within 2 of the model with the lowest AICc. We selected the most parsimonious model (lowest number of degrees of freedom) within that subset of models. In order to further confirm the importance of the predictors in the most parsimonious model, we also conducted model averaging using the model.avg() function in the MuMIn package. However, we performed this model averaging on simplified models with the same predictors but without interactions [41]. Statistical analyses were performed in program R v.4.2.2 (R Core Team 2023). We plotted the marginal effects of the most parsimonious models using the plot_model() function in the sjPlot v.2.8.10 R package [42].

## 3. Results

Antibodies to *T. gondii* were found in 48.7% (96/197; 95% CI: 41.8–55.7%) of the analysed raccoons and in 28.1% (25/89; 95% CI: 19.8–38.2%) of the raccoon dogs (Table 1). There was a significant difference in the prevalence estimates between both the species (*χ*^2^ = 9.87, df = 1, *p* = 0.002). In the case of the raccoon, positive results were recorded at titers 1:25 (39.5%), 1:50 (34.4%), 1:100 (16.7%) and 1:500 (9.4%), while positive results were obtained for the raccoon dog at titers 1:25 (48.0%), 1:50 (28.0%), 1:100 (12.0%) and 1:500 (12.0%).

**Figure 2:**
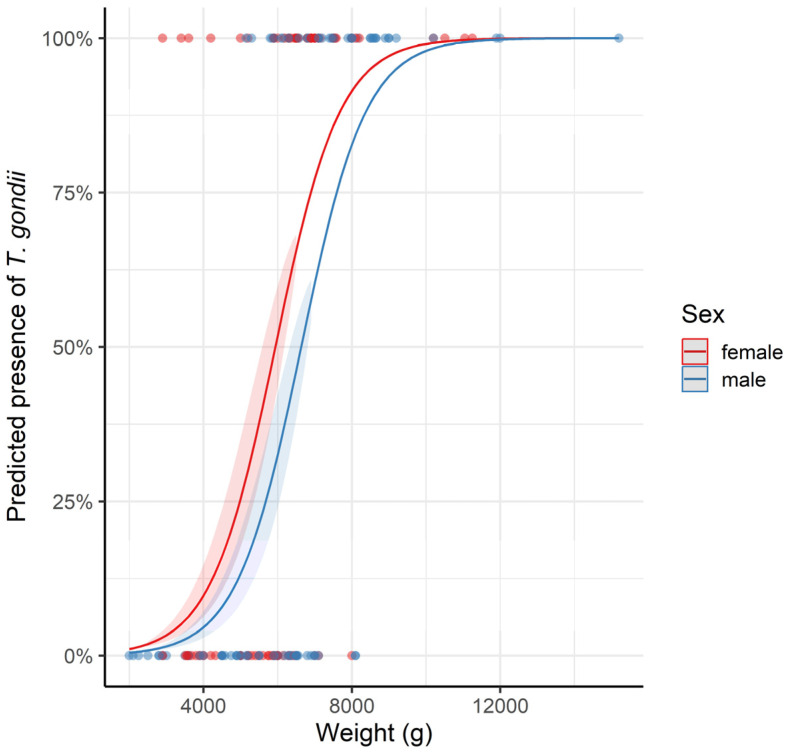
Marginal effects plot of a logistic regression model predicting the presence of *Toxoplasma gondii* antibodies in raccoons (*Procyon lotor*) from Zgorzelec County, Poland, as a function of weight and sex of the host. The 95% confidence intervals are shown and the plot is based on the most parsimonious model identified after model selection (see Table 2).

Since VIF values were ≤3.39 in all full GLMMs (without interactions) across both the species, our models did not have multicollinearity issues. When trying to identify predictors for the presence of *T. gondii* antibodies in raccoons, our model selection procedure resulted in seven equivalent models based on the AICc. According to the most parsimonious model, heavier hosts were more likely to be seropositive, as were females compared to males (Table 2, Figure 2). The terms of the most parsimonious model were included in the seven equivalent models. Model averaging performed after a model selection procedure on models without interactions confirmed the importance of both the predictors (Table S1). The marginal *R*^2^ of 0.592 indicated that the most parsimonious model had reasonably high power to predict accurately the presence of *T. gondii* antibodies in raccoons based on our fixed-effects predictors.

When trying to identify predictors for the presence of *T. gondii* antibodies in raccoon dogs, our model selection procedure resulted in seven equivalent models based on the AICc.

**Figure 3:**
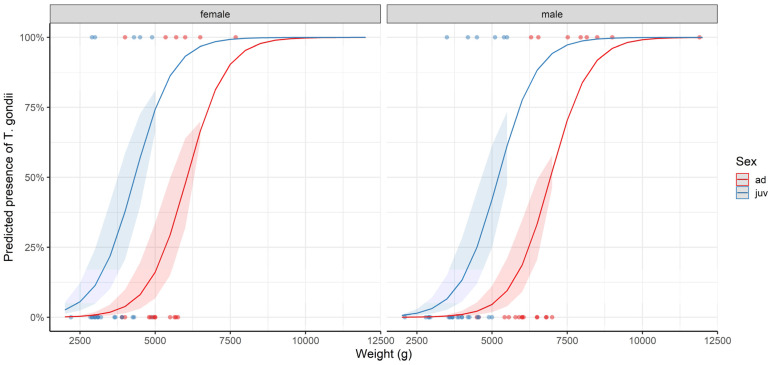
Marginal effects plots of a logistic regression model predicting the presence of *Toxoplasma gondii* antibodies in raccoon dogs (*Nyctereutes procyonoides*) from Zgorzelec County, Poland, as a function of weight, age category and sex of the host. The 95% confidence intervals are shown and the plot is based on the most parsimonious model identified after model selection (see Table 2).

According to the most parsimonious model, heavier hosts were more likely to be seropositive, as were females compared to males. Moreover, juvenile raccoon dogs were more likely to be seropositive than adults (Table 2, Figure 3). The terms of the most parsimonious model were included in the seven equivalent models (Table S1). When considering models without interactions (in order to perform model averaging), the model selection procedure gave rise to a single best-supported model. This model was identical to the most parsimonious model. The most parsimonious model had a marginal *R*^2^ of 0.488.

## 4. Discussion

In the raccoon’s native range, seroprevalence of *T. gondii* ranged from 13 to 92% [43,44,45,46,47,48,49,50]. In contrast, the seroprevalence values reported from Asia and Europe, where the raccoon was introduced, were between 0 and 65.5% [30,33,36,51,52,53]. A comparison of the results of different studies is usually difficult; however, as the determination of the serostatus can be carried out using blood or meat juice and can be based on different tests, such as the MAT, the direct agglutination test (DAT), the indirect hemagglutination test (IHAT), the latex agglutination test (LAT) or an enzyme-linked immunosorbent assay (ELISA). Experimental studies on raccoons have shown that the MAT has a higher sensitivity and reaction speed compared to the IHAT and LAT [35], whereas the ELISA, tested on pig sera, provided comparable results to the MAT [54]. Consequently, subsequent studies examining the seroprevalence of *T. gondii* antibodies in raccoons have mostly employed the MAT test [33,36,45,46,47,49]. Moreover, previous experimental studies have demonstrated the validity of serological analyses using the MAT, as viable *T. gondii* can be recovered from isolates from a large number of seropositive animals. In these studies, a high rate of isolated viable parasites was found to be positively associated with the MAT titre [55,56,57]. In the past, the MAT with a cut-off value of 1:25 was frequently used to diagnose *T. gondii* infections in a variety of domestic and wild animal species [1,58,59,60], including raccoons [33,36]. The accuracy of the seroprevalence estimates is strongly influenced by the sample size. If results from different studies are considered, especially if the sample size is small, the confidence intervals should be considered [53].

The seroprevalence for raccoons in the present study aligns with estimates from various regions in North America [45,50,61] and Central Europe [33,53], where seroprevalence commonly varies between 40 and 60%. The highest seroprevalence estimate from outside the raccoon’s original distribution area has been reported from Germany, where antibodies against *T. gondii* were found in 65.5% (61/93; 95% CI: 27.3–81.2%) of raccoons using the MAT on serum samples [36]. However, due to the smaller sample size, this estimate had wide 95% CIs. Lower prevalence values are reported from Luxembourg (19%; 95% CI: 2.3–35.8%) and the Czech Republic (0%; 95% CI: 0–19.5%) [33,62]. Similarly, the *T. gondii* seroprevalence in the only previous study of raccoons from Poland (based on meat juice and analysis with the ELISA) was lower (13.3%; 95% CI: 1.7–40.5) [30] than that observed in the present study. In comparison, a recent study from the neighbouring federal state of Saxony in Germany (based on meat juice and analysis with ELISA) was also based on a smaller number of samples (12/32) [53], but gave rise to a similar prevalence estimate as the present study (46.9%; 95% CI: 30.9–63.6%). It is clear that seroprevalence value can vary between regions, as other authors have already pointed out [33,43,48,52,53].

In contrast to raccoons, studies on *T. gondii* infections in raccoon dogs are rare and mostly performed on animals from fur farms, particularly in China [63,64,65]. Serological studies on wild raccoon dogs have so far only been carried out in Poland and Denmark. The seroprevalence reported in the present study was comparable to the estimate obtained in an earlier study in the Głeboki Bród Forest District in the northeastern part of Poland, in which 25.0% of the tested animals had antibodies against *T. gondii* [28]. However, this study was based on only 12 animals and the ensuing 95% confidence intervals were thus large (8.5–53.7%). A significantly higher seroprevalence has been reported from Denmark, where 42.7% (95% CI: 36.2–49.5%) of the 227 raccoon dogs tested were seropositive, with seroprevalence showing greater regional variations (37.1–57.1%) [22].

The relationship between high seroprevalence and cat density has been a recurring topic. For example, the presence and frequency of domestic and feral cats increase the risk of infection for wildlife species [66]. In domesticated and wild ruminants (*Capreolus capreolus* and *Ovis orientalis musimon*) and wild boar (*Sus scrofa*), the presence of domestic cats and wild cats is considered a major risk factor for *T. gondii* infection [58,59,60,67,68]. Hancock et al. [47] and Heddergott and Müller [36] associated the high prevalence in raccoons in their study areas with the high density of cats and the associated high oocyst excretion. The low seroprevalence values determined in raccoons in Japan (<15%) support this assumption, as the country has a low density of domestic cats [52,69,70] and wild cat species are absent from the main islands. According to Yamaguchi et al. [70], the highest prevalence of *T. gondii* antibodies was found in raccoons that share their habitat with feral domestic cats (*Felis catus*).

According to local hunters and farmers, there is a very high density of free-ranging and feral domestic cats in the entire area of Zgorzelec County, while wild cat species such as the European wildcat (*Felis s. sylvestris*) and the lynx (*Lynx lynx*) do not occur in this area [71,72]. In this context, the significant difference in the seroprevalence of the two carnivore species tested in the present study area is difficult to assess. This could be due more to the fact that despite the omnivorous lifestyle, the diet of the two species in the study area is very different and the food components are infected with these parasites to varying degrees. In general, the prey of both raccoons and raccoon dogs are known to transmit *T. gondii* cysts [1]. Feeding ecology studies on raccoons and raccoon dogs are extremely rare in Central Europe. Two dietary studies in neighbouring eastern Germany found that the main dietary components of raccoons are earthworms, molluscs and fruits [73,74], while the most commonly eaten components of raccoon dogs are invertebrates, small mammals, amphibians and reptiles [75]. It is likely that the individual food components are infected with *T. gondii* to varying degrees, which explains the differences in seroprevalence between the two species.

Our results showed that body weight had a strong influence on the serostatus of both raccoons and raccoon dogs. Heavier animals were more likely to be seropositive than animals with a lower body weight. In general, the body weight of the raccoon increases with age [76] and can therefore be used as an indicator of age, which can also be applied to the raccoon dog. Age has been described as a risk factor for *T. gondii* infection, as the probability of contact with the parasite increases with time [77]. Most studies only consider two age classes, juvenile and adult, and body weight may be a better indicator than the age of the animal. Consequently, the studies that considered both weight and age categories in their analysis found an effect of weight, but not of age [33,36,52,53]. In contrast, studies that have reported an increased likelihood of the presence of *T. gondii* antibodies in older raccoons did not simultaneously test for the effect of weight [43,45,46,49,78]. In the case of the raccoon dog, for a given weight, juveniles were more likely to be seropositive than adults. This result is slightly counterintuitive as the probability of contact with the parasite likely increases with time. Juveniles may thus have an increased likelihood of encountering the parasite, as their autumnal home ranges can be larger than those of adults and juveniles disperse in late summer/early spring [79]. In addition, we cannot definitively rule out the possibility that the outcome is influenced by our relatively small sample size.

Our results show an association between the presence of *T. gondii* antibodies and sex in both the species, with females more likely to be seropositive than males. In contrast, studies on German raccoons [33,36] and Danish raccoon dogs [22] did not find any effect of sex on the likely presence of *T. gondii* antibodies. However, in a recent German study, male raccoons were significantly more likely to be seropositive than females [53]. The authors suspected a connection with a lower need for security and a larger home range of the males, which is ultimately related to a different diet than that of the females. These authors analysed raccoons harvested for their fur. Their specimens were thus not a random sample of the population, as heavier males, which are more likely to be seropositive, are preferentially harvested for their fur. However, Hwang et al. [49] also found a higher seroprevalence in male raccoons in North America compared to juveniles of both sexes and surmised a connection with the higher mobility and greater home ranges. Further research on both host species is clearly needed to improve our understanding of the effect of sex on the presence of *T. gondii* antibodies and the underlying reasons for any differences between the sexes.

The results of the present study show that *T. gondii* is widely distributed in the environment of Zgorzelec County, Poland. Although the raccoon and raccoon dog do not contribute significantly to the epidemiology of this parasite, they may nevertheless contribute to the maintenance of this parasite in the ecosystem. The improper disposal of infected carcasses resulting from hunting and traffic accidents can inadvertently provide food for feral domestic cats, which subsequently may excrete *T. gondii* oocysts into the environment through their faeces [33]. In contrast to some regions in Germany where raccoon meat is consumed by humans [33], we know of no incidence of raccoon or raccoon dog meat being eaten in the study area. Furthermore, general hygiene guidelines should be followed in fur production. For this reason, the direct risk of infection for humans is low.

## 5. Conclusions

The aim of the present work was to test for the first time the seroprevalence of *T. gondii* infection in two free-living invasive carnivores from Zgorzelec County, southwestern Poland. The high seroprevalence estimate indicate a high degree of circulation of this parasite in the regional ecosystem. Although both carnivores do not play a major role in the epidemiology of *T. gondii*, they may still contribute to the maintenance of the parasite in the ecosystem. Continued investigation and monitoring of both invasive and native predatory mammals, including their food components, are warranted and necessary to further clarify the occurrence, prevalence and epidemiology of this protozoan.

## Figures and Tables

**Table 1:** Seroprevalence of *Toxoplasma gondii* in raccoons (*Procyon lotor*) and raccoon dogs (*Nyctereutes procyonoides*) by sex, age and collection year.

		*Procyon lotor*			*Nyctereutes procyonoides*		
Variable	Category	No. Tested	No. Positive	Prevalence in % (95% CI) ^1^	No. Tested	No. Positive	Prevalence in % (95% CI)
Gender	Male	100	45	45.00 (35.63–54.76)	49	14	28.57 (17.83–42.55)
	Female	97	51	52.58 (42.74–62.21)	40	11	27.50 (16.06–43.03)
Age	Juvenile	81	27	33.33 (24.04–44.20)	50	11	22.00 (12.67–35.48)
	Adult	116	69	59.48 (50.37–67.96)	39	14	35.90 (22.76–51.66)
Collection year	2019	108	53	49.07 (39.85–58.37)	38	10	26.32 (14.91–42.23)
	2020	89	43	48.31 (38.23–58.54)	51	15	29.41 (18.70–43.12)
Total		197	96	48.73 (41.85–55.67)	89	25	28.09 (19.81–38.26)

^1^ CI—confidence interval.

**Table 2:** Logistic regressions identifying predictors for the presence of *Toxoplasma gondii* antibodies in (**a**) raccoons (*Procyon lotor*) and (**b**) raccoon dogs (*Nyctereutes procyonoides*) from Zgorzelec County, Poland. Results are presented for the most parsimonious model identified after model selection. In the initial model, we included sex, age (juvenile vs. adult), weight and year of sampling as fixed factors. We only included two-way interactions.

Coefficients	Estimate	s.e.	z-Value	*p*-Value
(**a**)				
(Intercept)	−6.8193	1.1125	−6.130	<0.0001
Weight	0.0012	0.0002	6.425	>0.0001
Sex: Males	−0.8016	0.3685	−2.175	0.0296

(**b**)				
(Intercept)	−9.4400	2.3071	−4.092	−4.092
Weight	0.0016	0.0004	3.937	3.937
Sex: Males	−1.3802	0.6614	−2.087	−2.087
Age: Juvenile	2.7118	0.9695	2.787	2.787

In the case of the continuous predictor variable (weight), the logistic regression coefficient gives the change in the log odds of seroprevalence for a one-unit increase in weight. In the case of the categorical variables, the logistic regression coefficient gives the change in the log odds of seroprevalence when considering males and juveniles relative to females and adults, respectively.

## Data Availability

The data presented in this study are available upon request from the corresponding author.

## Supplementary Materials

The following supporting information can be downloaded at: https://www.mdpi.com/article/10.3390/pathogens13030210/s1; Table S1: Logistic regressions identifying predictors for the presence of *Toxoplasma gondii* antibodies in (a) raccoons (*Procon lotor*) and (b) raccoon dogs (*Nyctereutes procyonoides*) from Zgorzelec County, Poland. Results are presented for the most parsimonious model identified after model selection. In the initial model, we included sex, age (juvenile vs. adult), weight and year of sampling as fixed factors. We only included two-way interactions.
