# Supplementary material for: Prevalence of Toxoplasma gondii Antibodies and Risk Factors in Two Sympatric Invasive Carnivores (Procyon lotor and Nyctereutes procyonoides) from Zgorzelec County, Poland
Source: Pathogens. 2024 Feb 28;13(3):210. doi: 10.3390/pathogens13030210 (PMC10975512; doi:10.3390/pathogens13030210)
Supplement: Supplementary file 1 [file pathogens-13-00210-s001.zip › pathogens-2810469-supplementary.pdf]

Table S1. Logistic regressions identifying predictors for the presence of Toxoplasma gondii antibodies in *raccoons* from Zgorzelec County, Poland. (**a**) Presented are results of model averaging (full averages) performed on the (**b**) subset of best models obtained after performing a model selection procedure on models without interactions. In the initial model, we included sex, age (juvenile vs. adult), weight and year of sampling as fixed factors. The subset of best models is sorted following ascending AICc values, with the most parsimonious model in bold.

(a)

Coefficients	Estimate	adj. s.e.	z value	p value
(Intercept)	−7.3148	1.3209	5.538	<0.0001
Weight	0.0012	0.0002	6.048	<0.0001
Sex-Male	−0.7952	0.3730	2.132	0.033
Age-Juvenile	0.3029	0.4356	0.696	0.164
Year of sampling-2020	0.0513	0.2118	0.242	0.623

(b)

combination	df	logLik	AICc	ΔAICc	weight
2+4	3	−93.780	193.7	0.00	0.363
1+2+4	4	−92.766	193.7	0.06	0.353
2+3+4	4	−93.670	195.5	1.87	0.143
1+2+3+4	5	−92.628	195.6	1.89	0.141

Term Codes: 1: Age; 2: Weight; 3: Year of sampling; 4: Sex
